# Clinicopathologic and prognostic significance of FOLR1 expression in gastric adenocarcinoma and its association with PD-L1, HER2, MMR, and p53 status

**DOI:** 10.1007/s00428-026-04455-9

**Published:** 2026-02-27

**Authors:** Ahmet Erbağcı, Hatice Şeneldir, Tuçe Söylemez Akkurt, Şurahbil Yağbasa, Ayşe Nur Toksöz Yıldırım

**Affiliations:** 1 Department of Pathology, Istanbul Medeniyet University, Goztepe Prof.Dr.Suleyman Yalcin City Hospital, Eğitim Mah. Fahrettin Kerim Gökay Caddesi, Kadıköy, Istanbul 34722 Turkey; 2 Department of General Surgery, Istanbul Medeniyet University, Goztepe Prof.Dr.Suleyman Yalcin City Hospital, Eğitim Mah. Fahrettin Kerim Gökay Caddesi, Kadıköy, Istanbul, 34722 Turkey

**Keywords:** Gastric adenocarcinoma, FOLR1, HER2, PD-L1, MMR

## Abstract

Gastric adenocarcinoma carries high mortality despite modern multimodal therapy. Reliable biomarkers that refine prognosis and suggest therapeutic strategies are needed. We evaluated the clinicopathologic and prognostic significance of folate receptor-alpha (FOLR1) expression in a large single-institution cohort. We retrospectively analyzed 259 gastrectomy specimens (2016–2025). Immunohistochemistry included FOLR1 (H-score 0–300), HER2, PD-L1 (CPS; positive if ≥ 1), p53 status, and MMR proteins (MLH1/MSH2/MSH6/PMS2). The FOLR1 cut-off for recurrence-free survival (RFS) was derived by Receiver operating characteristic(ROC) analysis. Associations were tested with Chi-square or Fisher’s exact tests and Spearman correlation; survival was assessed by Kaplan–Meier and Cox regression. ROC for RFS yielded an area under the curve (AUC) of 0.653 (95% CI 0.578–0.728; *p* < 0.001) and defined a FOLR1 H-score cut-off of 40; ROC for overall survival (OS) was not significant (AUC 0.528; *p* = 0.443). Using this threshold, FOLR1-high tumors (≥ 40; *n* = 48) showed significantly shorter RFS (mean 24.1 vs 266.6 months; log-rank *p* < 0.001) and shorter OS (mean 32.4 vs 53.9 months; p = 0.047) than FOLR1-low tumors. On univariate analysis, FOLR1-high predicted inferior RFS (HR 4.15; 95% CI 2.67–6.47; *p* < 0.001); in multivariate Cox modeling it remained an independent predictor (HR 1.99; 95% CI 1.15–3.45; *p* = 0.015). FOLR1-high status associated with advanced T stage (*p* = 0.045), vascular invasion (*p* < 0.001), higher M1 frequency (*p* < 0.001), and proximal location (*p* = 0.038). It correlated with PD-L1 positivity (*p* < 0.001; FOLR1 H-score vs PD-L1 CPS Spearman r = 0.286; *p* < 0.001), HER2 positivity (*p* < 0.001), and p53 mutant pattern (*p* < 0.001), and was inversely associated with MMR deficiency (*p* = 0.010). N-stage distribution was not different overall, although a linear trend toward higher nodal stage with FOLR1-high was observed (*p* = 0.013). No significant associations were found with Lauren or WHO subtype, histologic grade, lymphatic or perineural invasion, extracapsular extension, neoadjuvant therapy, age, or sex. High FOLR1 expression identifies a biologically aggressive subset of gastric adenocarcinoma with inferior RFS and independent prognostic value. Its concurrent association with PD-L1 and HER2, together with relative absence in dMMR tumors, supports biomarker-guided strategies that consider FOLR1 alongside HER2 and PD-L1, and provides a rationale to explore FOLR1-targeted therapies in selected patients.

## Introductıon

Gastric adenocarcinoma remains one of the leading causes of cancer-related mortality worldwide. Despite advances in multimodal therapy, including gastrectomy, systemic chemotherapy, and the use of targeted agents, the prognosis for advanced disease remains poor, underscoring the continuing need for reliable prognostic biomarkers and novel therapeutic targets [1, 2].

The folate receptor alpha (encoded by the FOLR1 gene) is a high-affinity folate-binding cell-surface glycoprotein anchored via a glycosylphosphatidylinositol (GPI) moiety [3]. Under physiologic conditions, its expression is limited to certain epithelial tissues, but it is frequently overexpressed in a variety of epithelial malignancies including ovarian, lung, breast, kidney, and endometrial carcinomas, making it an attractive molecular target [4–6]. Beyond facilitating folate uptake, FOLR1 overexpression is implicated in enhanced proliferative capacity, anti-apoptotic signaling, and tumor aggressiveness. Recently, the antibody–drug conjugate mirvetuximab soravtansine-gynx (Elahere®) received full approval from the U.S. Food and Drug Administration for the treatment of platinum-resistant, FOLR1-positive epithelial ovarian cancer, highlighting the clinical relevance of FOLR1 as a therapeutic biomarker [7].

Data on FOLR1 expression in gastric carcinoma remain limited, and its clinicopathologic relevance is incompletely characterized. While some systematic reviews suggest that high FOLR1 expression correlates with poor survival across solid tumors, its associations with tumor biology, underlying molecular subtypes, and clinically actionable biomarkers in gastric cancer have not been systematically evaluated [8, 9]. In particular, it is unclear whether FOLR1 expression relates to HER2, PD-L1, and mismatch repair status, or corresponds to distinct prognostic subsets. This study aimed to evaluate FOLR1 immunohistochemical expression in a large cohort of gastric adenocarcinoma patients, to correlate it with histologic and molecular features, and to assess its prognostic relevance with respect to RFS and OS.

## Materials and methods

### Case selection and clinicopathologic evaluation

This retrospective study included 259 patients diagnosed with gastric adenocarcinoma between January 2016 and April 2025 at Istanbul Medeniyet University,Goztepe Prof.Dr.Suleyman Yalcin City Hospital. Representative formalin-fixed, paraffin-embedded (FFPE) tumor blocks were selected from the pathology archives. All hematoxylin and eosin (H&E) slides were reviewed to confirm the diagnosis and to classify the tumors according to the 2019 World Health Organization (WHO) Classification of Digestive System Tumours and the Lauren classification.

Clinicopathologic data including age, sex, tumor localization, histologic grade, T/N/M stage, lymphatic, vascular, perineural, and extranodal invasion, as well as treatment history, were recorded.

### FOLR1 immunohistochemistry

Immunohistochemistry for FOLR1 was performed using a Leica Bond III automated stainer (Leica Microsystems, Wetzlar, Germany). Four-micrometer FFPE sections were subjected to heat-induced epitope retrieval for 20 min with Leica Epitope Retrieval Solution 2, followed by incubation with the FOLR1 mouse monoclonal antibody (clone BN3.2, Novocastra, Leica Microsystems; catalogue no. NCL-F-FRalpha) at a 1:50 dilution for 15 min [10]. Detection was performed using a polymer-based DAB system, and hematoxylin was used for counterstaining. Fallopian tube epithelium was used as a positive control tissue, and appropriate negative controls were included in each staining run.

### Other ancillary studies

For the remaining markers, 4-μm FFPE sections were deparaffinized, rehydrated, and subjected to heat-induced epitope retrieval in citrate buffer (pH 6.0) or EDTA buffer (pH 9.0), depending on antibody requirements. Endogenous peroxidase activity was blocked with 3% hydrogen peroxide. The primary antibodies used were HER2 (clone 4B5, Ventana), PD-L1 (clone 22C3, Dako), p53 (clone DO-7, Ventana), and mismatch-repair proteins MLH1 (ES05, Leica), MSH2 (79H11, Leica), MSH6 (EP49, Leica), and PMS2 (EP51, Dako). No PCR- or NGS-based MSI testing was performed; therefore, dMMR was used as a surrogate for presumed MSI-H status. EBV status was assessed by EBER in situ hybridization (ISH) on representative FFPE sections using the Leica Bond III platform. Detection was performed using a polymer-based DAB system, and hematoxylin was used for counterstaining. Appropriate positive and negative controls were included in each staining run.

### Evaluation of ımmunostaining

FOLR1 expression was evaluated semi-quantitatively based on the percentage of tumor cells showing membranous staining and the intensity of that staining. An H-score was calculated by multiplying the staining intensity (0, 1 +, 2 +, 3 +) by the percentage of positive tumor cells (0–100), resulting in a total score ranging from 0 to 300. According to ROC curve analysis for RFS, a cutoff value of ≥ 40 was used to define FOLR1-high expression, while cases below this threshold were considered FOLR1-low.

HER2 expression was evaluated according to the established scoring system for gastric carcinoma (0, 1 +, 2 +, 3 +). All cases with scores of 2 + or 3 + were additionally assessed by fluorescence in situ hybridization (FISH) to confirm HER2 gene amplification. Cases were considered HER2 positive when confirmed amplification by FISH [11].

PD-L1 expression was evaluated using the Combined Positive Score (CPS), calculated as the number of PD-L1–positive cells (including tumor cells, lymphocytes, and macrophages) divided by the total number of viable tumor cells and multiplied by 100. Cases with a CPS of ≥ 1 were considered PD-L1 positive [12]. MMR status was defined as deficient (dMMR) when there was complete loss of nuclear staining for any of the four proteins in tumor cells with intact expression in internal controls [13]. EBV status was assessed by EBER ISH and interpreted as positive or negative based on nuclear staining in tumor cells [14].

P53 expression was evaluated immunohistochemically, with abnormal patterns defined as either diffuse, uniformly strong nuclear staining in ≥ 90% of tumor cells or complete absence of nuclear staining in the presence of internal positive controls within stromal and inflammatory cells. In contrast, wild-type p53 expression was characterized by heterogeneous nuclear staining of variable intensity within tumor cells [15]. All slides were independently reviewed by four pathologists blinded to clinical data, and discrepant cases were resolved by consensus.

### Statistical analysis

All statistical analyses were conducted using IBM SPSS Statistics for Windows, version 26.0 (IBM Corp., Armonk, NY, USA). Continuous variables, including age and FOLR1 H-score, were expressed as mean ± standard deviation (SD) or median (range), and compared using the Mann–Whitney U test.

Associations between categorical variables, including clinicopathologic features and biomarker status (PD-L1, HER2, p53, MMR), were analyzed using the Chi-square test or Fisher’s exact test as appropriate. Correlations between FOLR1 H-score and PD-L1 CPS were evaluated using the Spearman’s rank correlation test.

The optimal cut-off for FOLR1 H-score was derived using ROC analysis based on RFS and subsequently applied to OS for consistency. Because the H-score distribution was markedly zero-inflated, median and quartile-based dichotomization was not informative. Survival analyses for RFS and OS were performed using the Kaplan–Meier method and compared using the log-rank test. Cox proportional hazards models were applied for univariate and multivariate analyses. Variables with clinical relevance or *p* < 0.10 in univariate analysis were entered into multivariate models. Collinearity was evaluated using correlation matrices and variance inflation factors. The proportional hazards assumption was assessed using log–minus–log survival plots and was not violated. A *p* value < 0.05 was considered statistically significant.

## Results

### Clinicopathologic characteristics

A total of 259 patients with gastric adenocarcinoma who underwent proximal, distal, or total gastrectomy were included in this study. Among them, 48 (18.5%) showed high FOLR1 expression, while 211 (81.5%) were classified as low FOLR1. The median age of the cohort was 66.9 years (range: 28–94), with 177 males and 82 females. Tumors were located proximally (esophagogastric junction, cardia, or corpus) in 111 cases (42.9%) and distally (antrum or pylorus) in 148 cases (57.1%). According to the Lauren classification, 158 (61.0%) were intestinal, 63 (24.3%) diffuse, and 38 (14.7%) indeterminate type. Based on the WHO 2019 classification, 4 (1.5%) were papillary, 126 (48.6%) tubular, 19 (7.3%) mucinous, 62 (23.9%) poorly differentiated, 33 (12.7%) mixed adenocarcinomas and 15 (5.7%) medullary carcinomas. For tumor stage, 44 (17.0%) were classified as T1–T2 and 215 (83.0%) as T3–T4b. Nodal metastasis was present in 191 patients (73.7%) (N1–N3b), while 68 patients (26.3%) were node-negative (N0). Distant metastasis (M1) was identified in 34 (13.1%) cases. A total of 52 patients (20.1%) had received neoadjuvant chemotherapy, while 207 (79.9%) had not. The maximum follow-up period was 110 months (9 years). Regarding histopathologic characteristics, 8 (3.1%) tumors were well differentiated, 128 (49.4%) intermediate, and 123 (47.5%) poorly differentiated. Lymphatic invasion was detected in 184 (71.0%), and vascular invasion in 112 (43.2%) cases. Perineural invasion was present in 181 (69.9%), and extranodal extension in 114 (44.0%) tumors.

Regarding immunohistochemical (IHC) markers, PD-L1 positivity (CPS ≥ 1) was observed in 74 (28.6%) cases. HER2 IHC scores were 0 (*n* = 188), 1 + (*n* = 23), 2 + (*n* = 30) and 3 + (*n* = 18). FISH testing was performed on all 2 + and 3 + cases, and amplification was confirmed in 19 cases (7.3%). A total of 43 (16.6%) cases showed loss of mismatch repair proteins by immunohistochemistry. MLH1/PMS2 loss accounted for 97.7% of dMMR cases (42/43), while isolated MSH6 loss was identified in one case (2.3%). An aberrant p53 pattern was present in 125 (48.3%), while 132 (51.0%) showed a wild-type staining pattern. EBV positivity was identified in 11/259 patients (4.2%).

### FOLR1 expression in gastric adenocarcinoma

Immunohistochemical analysis of FOLR1 showed a variable pattern of membranous staining across tumor samples, and the evaluation was performed based on membranous staining intensity and extent. Based on the ROC-derived cut-off histoscore of 40, tumors were categorized as FOLR1-low (< 40) or FOLR1-high (≥ 40). Overall, 48 of 259 cases (18.5%) exhibited high FOLR1 expression. Staining was mainly localized to the luminal or glandular surfaces of neoplastic cells, whereas non-neoplastic gastric mucosa was consistently negative. No staining was identified in stromal, vascular, or inflammatory elements.

The mean FOLR1 histoscore for the entire cohort was 22.8 ± 56.2 (range 0–280), with a median of 0, indicating that most cases displayed low or absent expression.

### Prognostic significance of FOLR1 expression

**Fig. 1:**
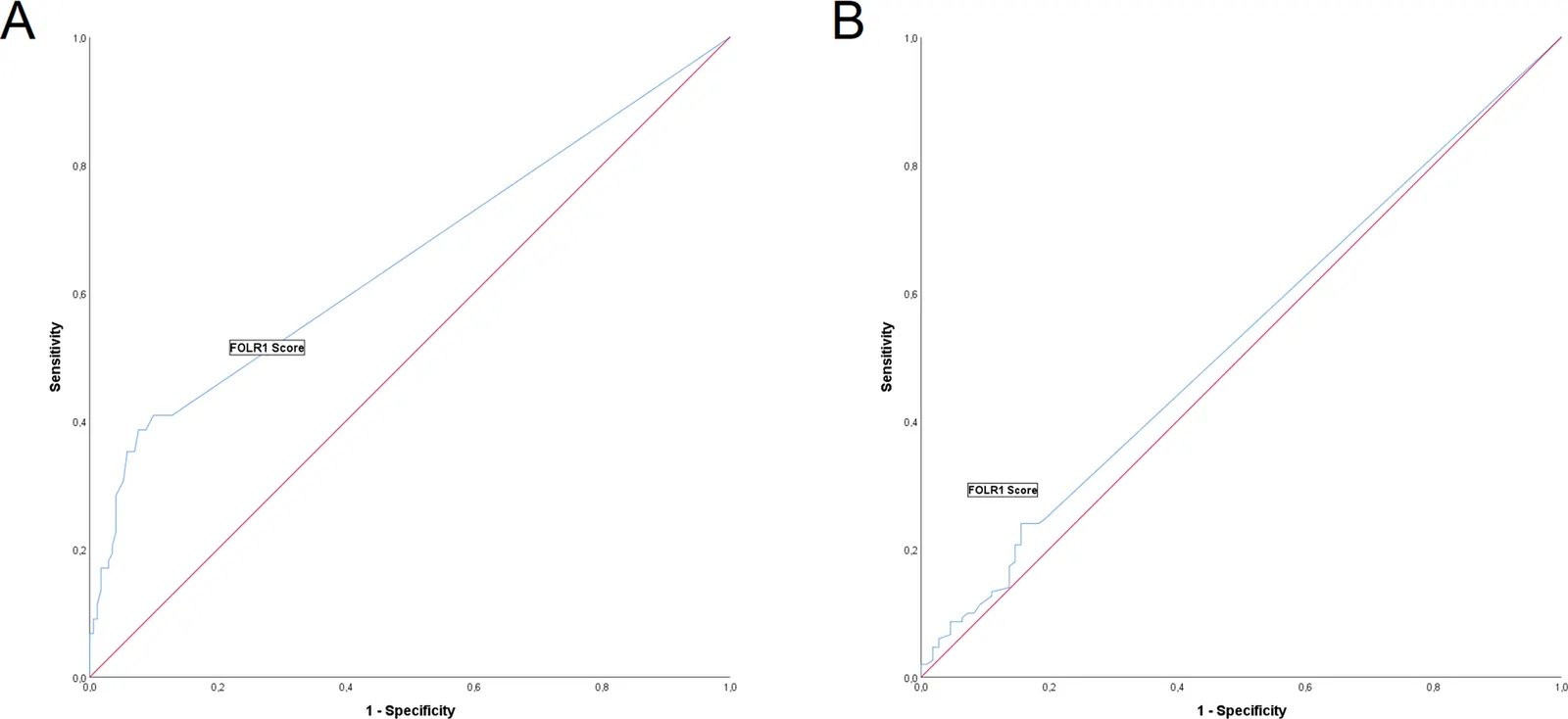
**A:** ROC curves for RFS according to FOLR1 histoscore. **B:** ROC curves for OS according to FOLR1 histoscore

**Fig. 2:**
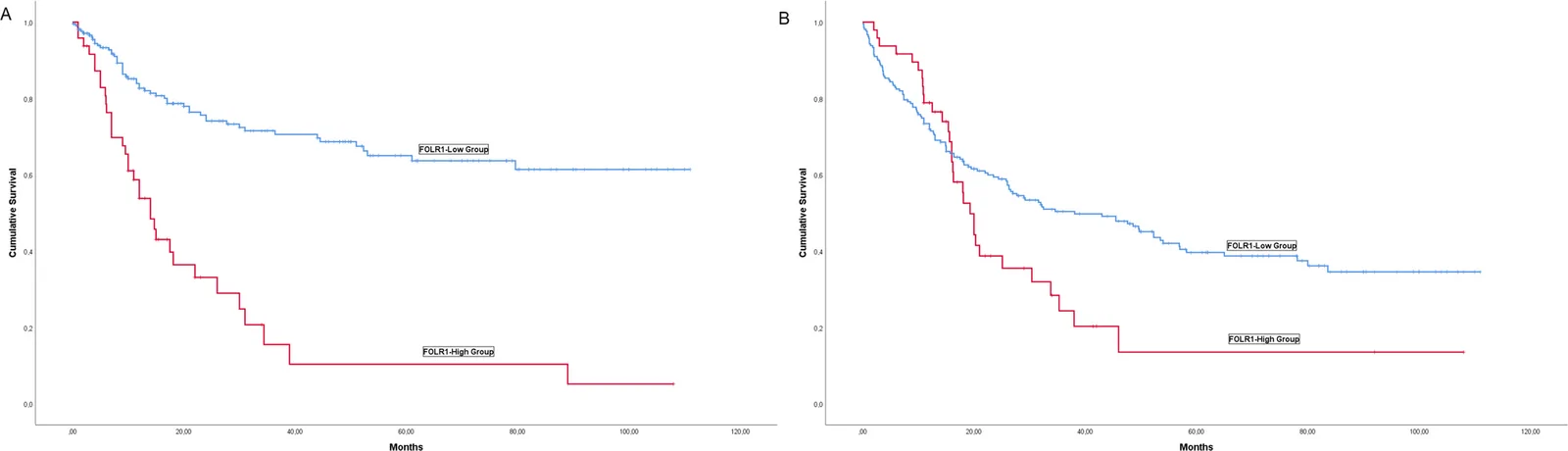
**A:** Kaplan–Meier survival analysis for RFS. **B:** Kaplan–Meier survival analysis for OS

ROC analysis was performed to determine the predictive value of FOLR1 histoscore for RFS and OS. For RFS, the AUC was 0.653 (95% CI 0.578–0.728, *p* < 0.001), indicating moderate discriminative capacity (Fig. 1A). The optimal cut-off value of 40 was used to define the FOLR1-high group. In contrast, the ROC curve for OS yielded an AUC of 0.528 (95% CI 0.457–0.599, *p* = 0.443), showing no significant predictive ability (Fig. 1B). During the 110-month follow-up, patients with FOLR1-high tumors exhibited significantly shorter RFS compared with those with FOLR1-low expression (median 14 months vs. not reached; log-rank *p* < 0.001). The estimated mean RFS was 24.1 months for FOLR1-high tumors (Fig. 2A).

Similarly, OS was reduced in the FOLR1-high group, with a median OS of 19.3 months (95% CI 16.5–22.1) compared with 38.1 months (95% CI 22.6–53.6) in the FOLR1-low group (log-rank *p* = 0.047) (Fig. 2B). Variables showing clinical relevance or *p* < 0.10 in univariate analysis were entered into multivariate Cox models. Collinearity was assessed using correlation matrices and variance inflation factors. The proportional hazards assumption was evaluated using log–minus–log survival plots for RFS and OS, with no major violations observed. In univariate Cox regression analysis, high FOLR1 expression was a significant adverse prognostic factor for RFS (HR 4.15; 95% CI 2.67–6.47; *p* < 0.001).

After adjustment for MMR status, T stage, N stage, and lymphatic invasion, FOLR1 remained an independent predictor of recurrence (HR 1.99; 95% CI 1.15–3.45; *p* = 0.015).

Higher T stage (*p* < 0.001) and lymphatic invasion (*p* = 0.023) were also independent adverse prognostic factors, while MMR deficiency correlated with improved outcome (*p* = 0.031).

### Clinicopathologic correlations

**Fig. 3:**
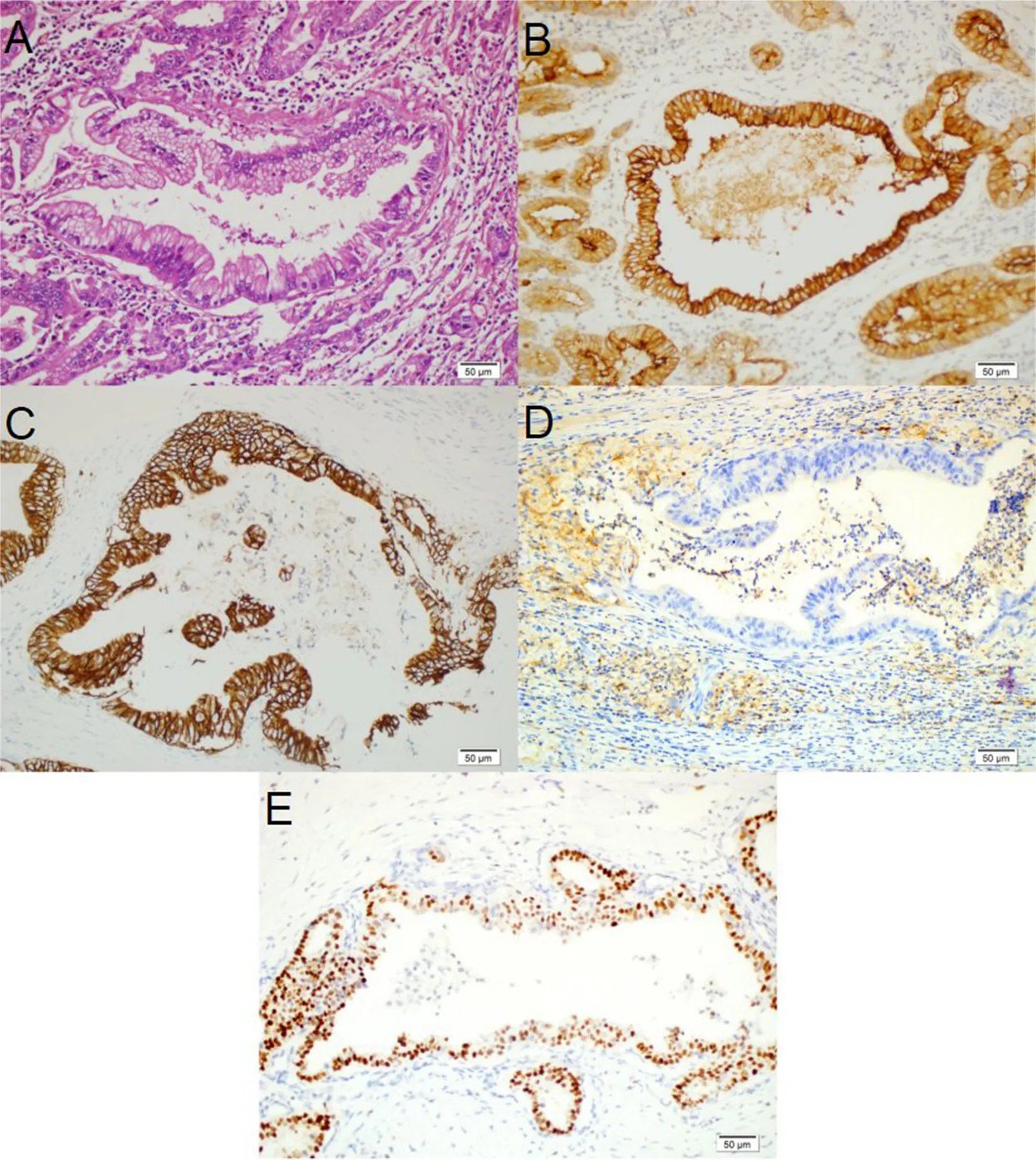
**A:** Intestinal-type gastric adenocarcinoma (H&E, × 200). **B:** Strong membranous FOLR1 expression (IHC, × 200). **C:** Strong membranous HER2 positivity (IHC, × 200). **D:** PD-L1 positivity (IHC, × 200). **E:** Strong nuclear p53 overexpression in tumor cells (IHC, × 200)

All clinicopathologic correlations with FOLR1 expression are summarized in Table 1. High FOLR1 expression showed strong positive associations with PD-L1 positivity (*p* < 0.001), HER2 positivity (*p* < 0.001), MMR proficiency (*p* = 0.010), and the p53 mutant pattern (*p* < 0.001). Among morphologic parameters, advanced T stage (T3–T4) (*p* = 0.045) and presence of vascular invasion (*p* < 0.001) were significantly correlated with high FOLR1 expression. A linear-by-linear association (*p* = 0.013) indicated a progressive increase in nodal involvement with increasing FOLR1 levels, and distant metastasis (M1) was also significantly more frequent among FOLR1-high cases (*p* < 0.001). Additionally, proximal tumor location was more common in the FOLR1-high group (*p* = 0.038). No significant associations were found between FOLR1 expression and Lauren or WHO histologic subtype, EBV status, histologic grade, lymphatic or perineural invasion, extracapsular nodal extension, neoadjuvant therapy, age, or sex Fig. 3.

**Table 1:** Clinicopathologic correlations of FOLR1 expression in gastric adenocarcinoma

Parameter	Category	FOLR1-low (*n* = 211)	FOLR1-high (*n* = 48)	*p*-value	Test
Age (years)	Median (range)	67(28-94)	66(40-84)	0.752	Mann–Whitney U
Sex	Male/Female	139/72	38/10	0.074	Chi-Square
Localization	Proximal/Distal	83/128	28/20	**0.038**	Chi-Square
Lauren Classification	Intestinal/Diffuse/Indeterminate	131/49/31	27/14/7	0.489	Chi-Square
WHO Histologic Type	Papillary/Tubular/Mucinous/Poorly diff./Mixed	1/105/18/48/39	3/21/1/14/9	0.385	Chi-Square
T Stage	T1–T2/T3–T4	42/169	2/46	**0.045**	Chi-Square
N Stage	N0/N1–3	62/149	6/42	**0.013**	Chi-Square
M Stage	M0/M1	201/10	24/24	** < 0.01**	Chi-Square
Neoadjuvant Chemotherapy	No/Yes	41/170	11/37	0.566	Chi-Square
Histologic grade	Well/Intermediate/Poor	8/103/100	0/25/23	0.385	Chi-Square
Lymphatic Invasion	Absent/Present	67/144	8/40	0.063	Chi-Square
Vascular Invasion	Absent/Present	135/76	12/36	** < 0.01**	Chi-Square
Perineural Invasion	Absent/Present	64/147	13/34	0.271	Chi-Square
Extranodal extension	Absent/Present	124/87	21/27	0.101	Chi-Square
PD-L1 Status	Negative/Positive	165/46	20/28	** < 0.01**	Chi-Square
HER2 Status	Negative/Positive	204/7	36/12	** < 0.01**	Chi-Square
MMR Status	Deficient/Proficient	169/42	47/1	**0.01**	Chi-Square
p53 Status	Wild-type/Abnormal	126/85	6/40	** < 0.01**	Chi-Square

FOLR1 expression was categorized as low (< 40) and high (≥ 40) based on ROC-derived histoscore cut-off. Chi-square and Mann–Whitney U tests were used for statistical comparison; *p* < 0.05 was considered significant

**Fig. 4:**
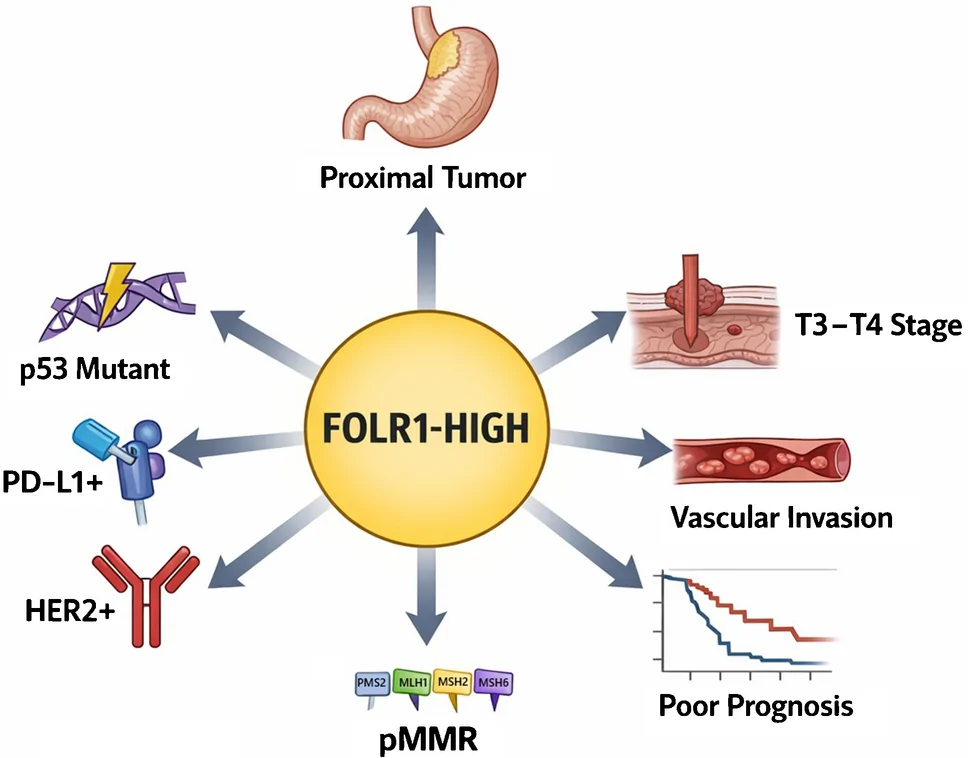
Schematic summary of the clinicopathologic and biomarker features associated with high FOLR1 expression in gastric adenocarcinoma. FOLR1-high tumors were enriched for proximal localization (58%), advanced T stage (T3–T4) (96%), vascular invasion (25%), PD-L1 positivity (42%, CPS ≥ 1), HER2 positivity (25%), abnormal p53 expression (85%) and pMMR status (98%), and were associated with poor survival

Taken together, these clinicopathologic and molecular associations define a FOLR1-high subset characterized by aggressive features and adverse prognosis (Fig. 4).

## Dıscussıon

FOLR1 is a GPI-anchored glycoprotein that mediates folate transport and is normally restricted to the apical surface of epithelial cells in organs such as the kidney, placenta, lung, and choroid plexus [16–18]. Its overexpression has been reported in several solid tumors, including pancreatic, triple-negative breast, lung, rectal, ovarian, and endometrial carcinomas, suggesting potential oncogenic and therapeutic relevance [9, 19–24]. The recent success of mirvetuximab soravtansine, an antibody–drug conjugate targeting FOLR1 in platinum-resistant ovarian and related carcinomas, has further emphasized its clinical importance. In this context, our study provides comprehensive evaluation of FOLR1 expression in gastric adenocarcinoma, exploring its associations with molecular markers and clinicopathologic parameters.

FOLR1 scoring varies among tumor types, and although semi-quantitative scoring is clinically used in ovarian carcinoma, no validated system exists for gastric cancer. We therefore adopted a histoscore approach to enable exploratory prognostic and statistical analyses in this setting.

FOLR1 overexpression was identified in 18.5% of gastric adenocarcinomas and was associated with proximal localization, advanced T stage, vascular invasion, and distant metastasis. These features indicate that FOLR1-high tumors represent an aggressive biological subset. Importantly, FOLR1 remained an independent prognostic factor for recurrence in multivariate analysis, underscoring its potential as a biomarker of poor outcome. Although further validation is required, the presence of a clinically actionable therapeutic pathway in other malignancies suggests that FOLR1 may represent a promising future target in gastric adenocarcinoma.

When compared with other tumor types (Table 2), several parallels and distinctions emerge. In pancreatic adenocarcinoma, FOLR1 positivity was an independent adverse prognostic factor for overall survival, consistent with our finding that FOLR1 independently predicted recurrence in gastric cancer [19].

**Table 2:** Comparison of FOLR1 expression patterns in gastric adenocarcinoma and other organ adenocarcinomas: antibody clone, cut-off values, sample size, and key clinicopathologic correlations

Study	Tumor Type/Organ	Sample Size	FOLR1 Antibody(Clone/Platform)	Scoring/Cut-off	%High FOLR1 Expression	Major Findings
Chen Y-L et al. 2012 [22]	Serous ovarian carcinoma	91	Western blot & RT-PCR (no IHC)	FOLR1 = ratio of FOLR1/GAPDH; High = > 1.0, Low ≤ 1.0	14%	High FOLR1 expression independently predicted poor outcomes:chemo-resistance and shorter survival
Omote et al. 2018 [18]	Pancreatic ductal adenocarcinoma	100	Mouse monoclonal anti-FOLR1 antibody (clone 26B3, Biocare Medical)	≥ 30% of tumor cells with membranous staining considered positive	37%	The FOLR1-positive group had a significantly poorer prognosis than the FOLR1-negative group. FOLR1 expression was an independent prognostic factor for overall survival
Zagorac et al. 2020 [19]	Triple-negative breast carcinoma	92	Polyclonal rabbit anti-FOLR1 antibody (ThermoFisher PA5-42004), Ventana BenchMark Ultra	H-score (0–300); < 10 negative, ≥ 10 positive; ROC-defined cut-off for recurrence > 75	85%	Increased relapse risk and predicted recurrence
Tamura et al. 2020 [21]	Lung adenocarcinoma	233	Anti-FOLR1 monoclonal antibody (clone 26B3, Eisai Co.)	H-score ≥ 60 = high	45%	High FOLR1 expression was significantly associated with EGFR mutation, lower PD-L1 expression, No correlation with overall or disease-free survival
Chen et al. 2022 [22]	Rectal adenocarcinoma	172	Mouse monoclonal anti-FOLR1 antibody, clone BN3.2 (Novocastra, Leica Microsystems)/platform not reported	Intensity-based score (0–4 +); high = 3 + –4 +, low = 0 + –2 +	42%	High FOLR1 expression significantly correlated with advanced post-treatment T and N stage vascular invasion, perineural invasion, and poor tumor regression after chemoradiotherapy. high FOLR1 expression predicted worse disease-specific survival and metastasis-free survival
McHenry et al. 2025 [24]	High-grade endometrial carcinoma with aberrant p53 expression	62 patients (66 cases, 291 TMA cores)	Ventana FOLR1-2.1 clone, predilute (Ventana Ultra platform)	% of tumor cells with moderate–strong membranous staining; ≥ 75% = high (Ventana companion diagnostic criterion)	8% (≥ 75% cut-off); 31% (≥ 25% cut-off)	Significant positive correlation between HER2 2 +/3 + status and FOLR1 positivity
Erbağcı et al.	Gastric adenocarcinoma	259	Mouse monoclonal anti-FOLR1 antibody, clone BN3.2 (Novocastra, Leica Microsystems)	H-score (0–300); ROC-defined cut-off for recurrence ≥ 40 accepted as high FOLR1 expression, < 40 low	18.5%	High FOLR1 expression rate associated with proximal site, advanced T stage, vascular invasion, and M1 Significant correlations: PD-L1 +, HER2 +, MMR-p, p53-aberrant Independent prognostic factor for recurrence Shorter RFS and OS in FOLR1-high group

*FOLR1* folate receptor alpha, *PDAC* pancreatic ductal adenocarcinoma; *TNBC* triple-negative breast carcinoma, *EGFR* epidermal growth factor receptor, *OS* overall survival; *RFS* recurrence-free survival, *MMR* mismatch repair. Cut-off values represent study-specific thresholds used to classify high versus low FOLR1 expression. “Major findings” summarize statistically significant clinicopathologic correlations and survival outcomes reported in each study

Similarly, in rectal adenocarcinoma, strong correlations between high FOLR1 expression and aggressive features such as vascular and perineural invasion, advanced T and N stage, and poor response to chemoradiotherapy [9]. These findings parallel the associations we observed with vascular invasion and advanced stage in gastric adenocarcinoma.

In triple-negative breast carcinoma, high FOLR1 H-scores predicted relapse and aggressive clinical behavior, comparable to the strong impact of FOLR1 on recurrence-free survival observed in our study [20].

In high-grade serous ovarian carcinoma (HGSOC), FOLR1 overexpression has been associated with chemoresistance, aggressive tumor behavior, and inferior survival outcomes [23]. Notably, although TP53 aberration is nearly universal in HGSOC, studies report that FOLR1-high tumors represent a particularly adverse subgroup with inferior outcomes. Likewise, in p53-mutantt high-grade endometrial carcinoma, FOLR1 positivity overlaps with HER2 overexpression, supporting our finding that FOLR1 expression in gastric tumors is frequently accompanied by HER2 positivity [24].

Conversely, lung adenocarcinoma demonstrates a more variable pattern, with FOLR1 expression linked to EGFR mutation and an immune-suppressive stroma but without a clear impact on survival. This variability suggests that the prognostic relevance of FOLR1 may depend on organ-specific molecular contexts [22].

Our study also highlights key molecular relationships. FOLR1-high tumors were significantly associated with HER2 and PD-L1 positivity and with MMR proficiency and p53 mutation pattern (Fig. 3). The inverse association with MMR deficiency is also biologically plausible: MMR-deficient gastric cancers generally have a better prognosis, and the predominance of MMR-proficient status among FOLR1-high tumors is consistent with their worse outcome [25]. The concomitant PD-L1 expression observed in our series parallels findings in ovarian carcinoma, where immune-evasion mechanisms are enriched in FOLR1-overexpressing tumors [5]. These observations collectively suggest that FOLR1 expression identifies a subset of gastric adenocarcinomas with oncogenic signaling activation, immune modulation, and poor clinical outcome.

EBV-associated gastric carcinoma has been recognized as a distinct molecular subset with characteristic histologic and clinical features. In our series, EBV positivity was identified in 4.2% of cases and was predominantly observed in the FOLR1-low group, without a significant association between EBV status and FOLR1 expression. Interpretation of these findings is limited by the small number of EBV-positive cases, and further studies with larger cohorts would be needed to clarify any potential relationships.

A notable finding of our study was the higher frequency of FOLR1-high tumors in the proximal stomach. Since proximal and esophagogastric junction adenocarcinomas generally exhibit poorer outcomes than distal types, this trend may indicate a potential, though not yet established, relationship between tumor localization and FOLR1 expression [26]. In addition, the observed association of FOLR1 with vascular invasion and metastasis may suggest a role in tumor progression, aligning with observations from pancreatic and rectal adenocarcinomas.

The co-occurrence of FOLR1 positivity with HER2 amplification, PD-L1 expression, MMR proficiency, and aberrant p53 in our cohort suggests that FOLR1-high tumors represent a proliferative phenotype with immune checkpoint engagement rather than an inflamed immune profile. This pattern contrasts with EBV-positive and MMR-deficient gastric cancers, which are typically immune-inflamed and clinically favorable, and supports the concept that FOLR1 expression clusters with aggressive, non-inflamed molecular contexts.

Similar associations have been reported in high-grade endometrial carcinoma, where FOLR1 expression overlaps with HER2 overexpression and aberrant p53, suggesting that FOLR1 may serve as a surrogate of high-grade tumor biology rather than functioning as an independent molecular driver [24]. The parallels between endometrial and gastric tumors in this regard highlight the possibility that FOLR1 positivity marks a metabolically demanding and clinically aggressive subset across multiple organ systems.

The variability in antibody performance and interpretation across studies, as demonstrated by recent comparative analyses of FOLR1 clones, underscores the need for methodological standardization [27]. Several limitations should be noted. This retrospective, single-center cohort consisted exclusively of surgically resected gastric cancers; therefore, patients presenting with unresectable or metastatic disease were not captured, introducing selection bias and potentially underestimating the impact of FOLR1, which was associated with poor outcomes in our series. Systemic treatment and treatment-response data were not consistently available, reflecting changes in therapeutic practice over time and limiting our ability to evaluate the predictive rather than prognostic value of FOLR1. Finally, the analysis relied on a single antibody clone and an RFS-optimized ROC cut-off, underscoring the need for validation in independent cohorts and prospective studies that include unresectable and metastatic cases to determine whether these molecular associations extend across the full clinical spectrum of gastric cancer.

## Conclusion

Our findings establish FOLR1 as a biomarker of adverse prognosis in gastric adenocarcinoma, associated with proximal localization, advanced stage, vascular invasion, and co-expression with HER2, PD-L1, and p53 mutation. In parallel with observations in pancreatic, rectal, ovarian, and endometrial carcinomas, these results support the role of FOLR1 as a marker of aggressive disease biology and a potential therapeutic target in gastric cancer.

## Data Availability

The datasets generated and/or analyzed during the current study are available from the corresponding author upon reasonable request.

## Declarations

### Permission to reproduce material

No previously published material, figures, or tables requiring copyright permission were reproduced in this manuscript.

### Clinical trial registration

Not applicable (this study is not a clinical trial).
